# Supplementary material for: An invariability-area relationship sheds new light on the spatial scaling of ecological stability
Source: Nat Commun. 2017 May 19;8:15211. doi: 10.1038/ncomms15211 (PMC5454453; doi:10.1038/ncomms15211)
Supplement: Supplementary Information — Supplementary Figures, Supplementary Notes and Supplementary References [file ncomms15211-s1.pdf]

Supplementary Figures

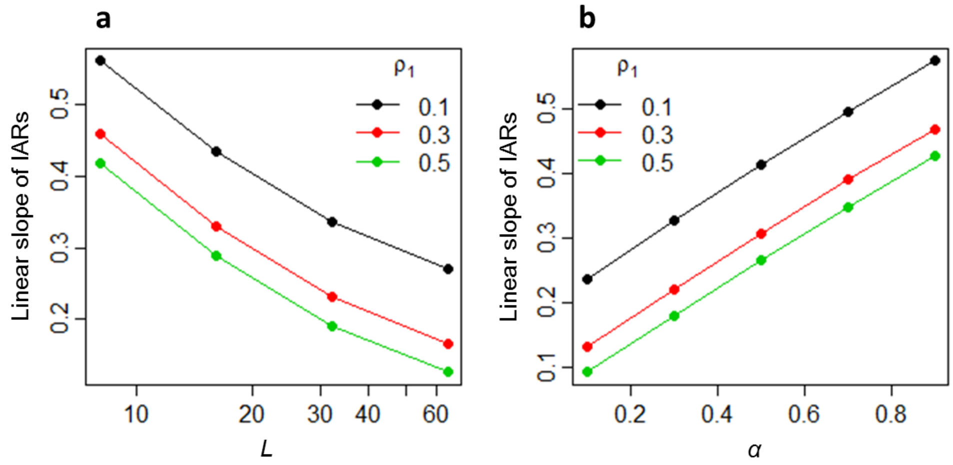

Supplementary Figure 1: IAR slope under exponential (a) and power (b) correlation-distance functions. The overall slope of IAR decreases with local correlation (ρ_1) and increases with the exponential decay rate ($1/L$) or the power-law exponent (α). The landscape consists of 128×128 grids. The IARs are constructed following the procedures described in the main text, and the slopes are from least-square fits between invariability and area on a log-log scale.

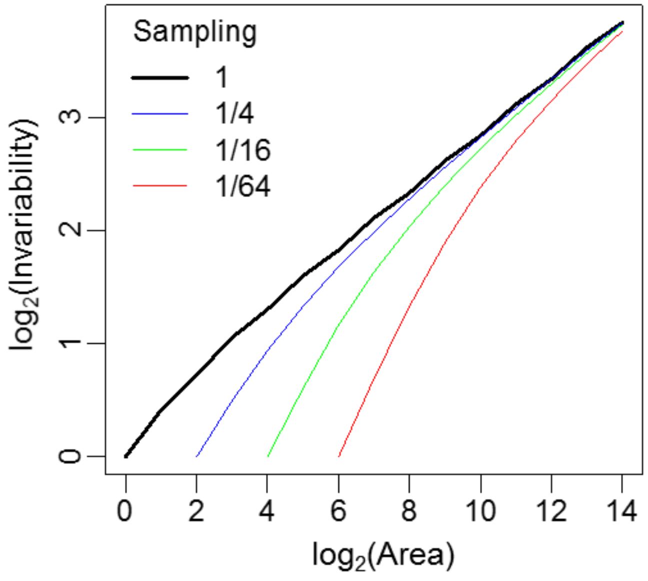

Supplementary Figure 2: IARs under incomplete spatial sampling. Black line corresponds to the IAR of the whole landscape. The colored lines correspond to IARs under different sampling intensities. Note the area (x-axis) is obtained by multiplying the number of sampled grids and the average “represented area” of each sample. For instance, when the sampling intensity is 1/4 of the whole landscape, each sampled grid is rescaled to represent an area of 4 grids. For each level of sampling intensities, we conduct 100 random samplings (i.e. randomly sample a corresponding proportion of grids from the whole landscape). The colored lines shows the average IARs across these 100 repeats.

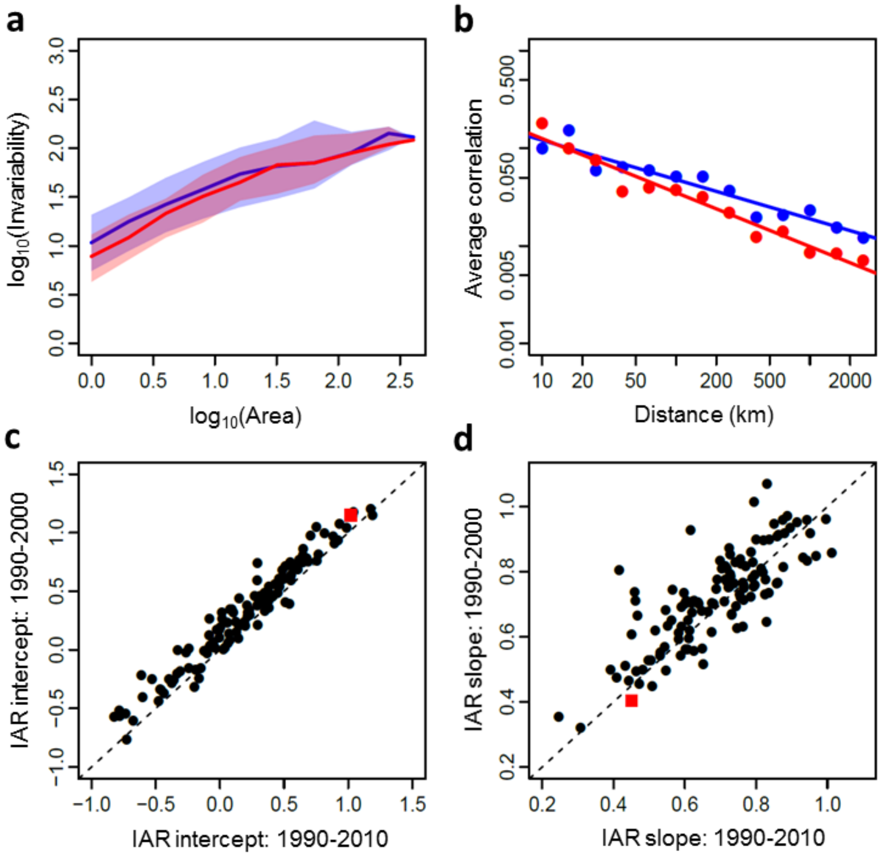

Supplementary Figure 3: The effect of observation length on IARs and correlation-distance relationships. (**a**) IARs of bird community biomass during 1990-2010 (red) and 1990-2000 (blue). Area is measured by the number of sampling routes (total number: 406). The lines show median invariability across 406 replicates, and the red shade shows 25% and 75% quantiles. (**b**) Correlation-distance relationships of bird community biomass during 1990-2010 (red) and 1990-2000 (blue). Points represent the average correlation of total community biomass corresponding to different distance categories. The red and blue lines are fitted power functions: $\rho(d) = 0.45\times d^{-0.55}$ (red) and $\rho(d) = 0.30\times d^{-0.40}$ (blue). (**c,d**) The intercept (**c**) and slope (**d**) of species-level IARs during 1990-2000 vs. 1990-2010. Each black point represents one bird species, and the red squares represent the values for community-level data.

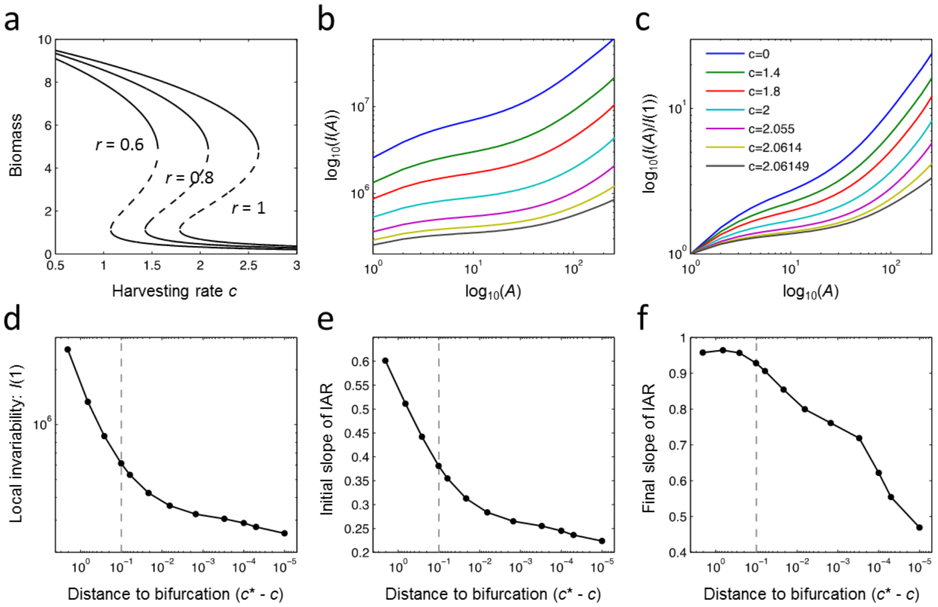

Supplementary Figure 4: Changes in IAR as the model metapopulation approaches a regime shift. (a) Equilibrium values of local population biomass as a function of the harvesting rate *c*, in the absence of dispersal and environmental perturbations. Local populations undergo a catastrophic shift from high biomass to low biomass as *c* increases beyond a tipping point. For instance, when the local growth rate *r* = 1, the tipping point occurs at the critical value *c** = 2.604. **(b)** IARs under different harvesting rates. For each value of *c*, we ran the metapopulation model to its dynamical equilibrium and calculated the invariability of biomass for different number of patches. Note that we plot up to 256 patches (i.e. half of the total *N*=512) because the largest pairwise distance in the ring landscape is 256. See panel (c) for colour codes. **(c)** Relative IARs under different harvesting rates. Relative IARs are obtained by dividing invariability at all scales by local invariability (*I*(1)). These relative IARs are convenient for comparing the shape and slope of IARs across scenarios. **(d)** Local invariability (*I*(1)) or the intercept of IAR as the system approaches the regime shift. The x-axis is the difference between the harvesting rate (*c*) and its critical value at the global tipping point (*c** = 2.0615) and decreases from left to right as the metapopulation gets closer and closer to the global tipping point. Local invariability decreases as *c* approaches its critical value. This decrease is faster away from the bifurcation (e.g. *c** - *c* > 0.1 or left of the dashed grey line). **(e)** Initial slope of IAR as the system approaches the regime shift. Initial slope is calculated by the log-log slope between areas 1 and 2. The initial slope decreases as *c* approaches its critical value. This decrease is faster away from the bifurcation (e.g. *c** - *c* > 0.1 or left of the dashed grey line). **(f)** Final slope of IAR as the system approaches the regime shift. The final slope is calculated by the log-log slope between areas 128 and 256. It decreases as *c* approaches its critical value. This decrease is faster closer to the bifurcation (e.g. *c** - *c* < 0.1 or right of the dashed grey line).

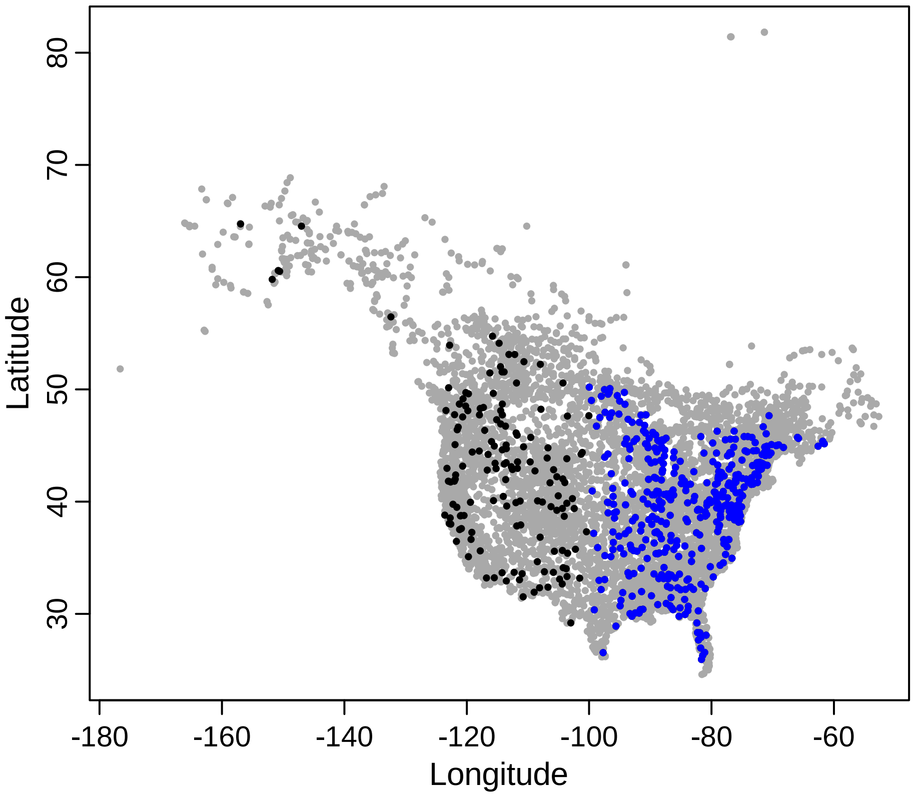

Supplementary Figure 5: Distribution of the sampling routes of North American Breeding Bird Survey. Grey points show all the routes that had been surveyed at least once since 1966. Black and blue points show 555 routes that have no missing records over the period 1990-2010. Among them, 406 routes (blue points) were located east of 100 ^{o}W, with an average distance of one route to its nearest neighbor ~ 50 km. The other 149 routes (black points) were located west of 100 ^{o}W, with an average distance ~ 100 km.

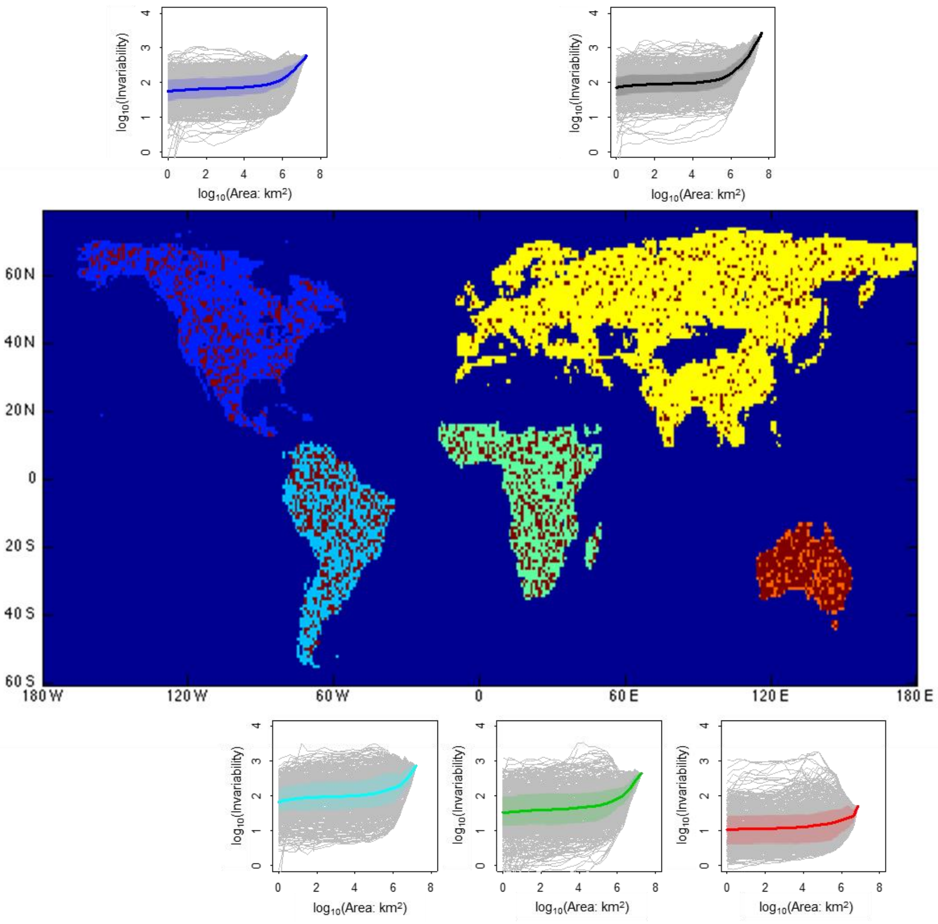

Supplementary Figure 6: IARs in the five continents. The map shows the five continents, and the red dots within them represent the randomly selected 500 starting points to generate IARs. The five inserted plots shows the resulting 500 IARs for respective continents, and the colored lines and shade represent the median and the 25% and 75% quantiles, respectively.

Supplementary Note 1: Theoretical analyses of the slope of IAR

Deriving the asymptotic slope of IAR

Here we derive the asymptotic slope (z_{asym}) of IAR by calculating the log-log slope of IAR as the area A goes to infinity:

$$z_{\text{asym}} = \frac{\ln I(A)}{\ln A} = \frac{\ln\left[I_1 \cdot \frac{A}{(A-1)\bar{\rho}_A+1}\right]}{\ln A} \cong -\frac{\ln(\bar{\rho}_A + A^{-1})}{\ln A} \quad \text{(S1)}$$

Eq. (S1) shows that, in order to obtain z_{asym}, we need to derive $\bar{\rho}_A$, i.e. the average pairwise correlation. Consider a square area $A = N^2$ (i.e. a group of grids on {1, 2, …, N} × {1, 2, …, N}), we have:

$$\bar{\rho}_A = \frac{1}{A(A-1)} \sum_{x,y\in A;x\neq y} \rho_{\text{x,y}} = \frac{1}{N^2(N^2-1)} \sum_{x_1=1}^{N} \sum_{y_1=1}^{N} \sum_{x_2=1}^{N} \sum_{y_2=1}^{N} \rho\left(\sqrt{(x_1-x_2)^2+(y_1-y_2)^2}\right)$$

$$= \frac{4}{N^2(N^2-1)} \sum_{k=1}^{N} \sum_{l=2}^{N} \left[(N+1-k)(N+1-l)\cdot\rho(\sqrt{(k-1)^2+(l-1)^2})\right] \quad \text{(S2)}$$

where $\rho(.)$ represents the correlation-distance relationship. Below we derive $\bar{\rho}_A$ and z_{asym} under two correlation functions.

(i) Exponential function: $\rho(d) = \rho_1 \times e^{-(d-1)/L}$

Under the exponential decay function, we have:

$$\bar{\rho}_A = \frac{4\rho_1}{N^2(N^2-1)} \sum_{k=1}^{N} \sum_{l=2}^{N} [(N+1-k)(N+1-l)\cdot e^{-\frac{\sqrt{(k-1)^2+(l-1)^2}-1}{L}}] \quad \text{(S3)}$$

Because $\frac{1}{\sqrt{2}}(x+y) \leq \sqrt{x^2+y^2} \leq x+y$ (when x, y > 0), we have:

$$\frac{4\rho_1}{N^2(N^2-1)} \sum_{k=1}^{N} \sum_{l=2}^{N} [(N+1-k)(N+1-l)\cdot e^{-\frac{(k+l-2)/\sqrt{2}-1}{L}}] \leq \bar{\rho}_A \leq \frac{4\rho_1}{N^2(N^2-1)} \sum_{k=1}^{N} \sum_{l=2}^{N} [(N+1-k)(N+1-l)\cdot e^{-\frac{k+l-3}{L}}] \quad \text{(S4)}$$

Denote $a = e^{-\frac{1}{L}}$, we can derive the right hand of Eq. S4 (recalling $A = N^2$):

$$Right\ hand\ of\ (S4) = \frac{4\rho_1 e^{\frac{3}{L}}}{N^2(N^2-1)} \left[N\cdot\frac{a}{1-a} + \frac{a^2(1-a^N)}{(1-a)^2}\right]\cdot\left[N\cdot\frac{a^2}{1-a} + \frac{a^2(1-a^N)}{(1-a)^2}\right] \cong \frac{4\rho_1 e^{\frac{3}{L}}}{N^2-1}\cdot\frac{a^3}{(1-a)^2} \triangleq \frac{C_1}{A}$$

where the approximate follows when N is large. Similarly, we can derive the left hand of Eq. S4:

$$Left\ hand\ of\ (S4)\ =\approx \frac{4\rho_1 e^{\frac{\sqrt{2}+1}{L}}}{N^2-1}\cdot\frac{b^3}{(1-b)^2} \triangleq \frac{C_2}{A}$$

where $b = e^{-\frac{1}{\sqrt{2}L}}$. Substituting $C_2A^{-1} \le \bar{\rho}_A \le C_1A^{-1}$ into Eq. S1, we have:

$$-\frac{\ln\left(C_1A^{-1}+A^{-1}\right)}{\ln A} \le z_{\text{asym}} \le -\frac{\ln\left(C_2A^{-1}+A^{-1}\right)}{\ln A} \quad \text{(S5)}$$

As A goes to infinity, the left and right hands of Eq. S5 both converge to 1. Thus, $z_{\text{asym}} = 1$.

(ii) Power function: $\rho(d) = \rho_1 \times d^{-\alpha}$

Under the power-decay function, we have:

$$\bar{\rho}_A = \frac{4\rho_1}{N^2(N^2-1)}\sum_{k=1}^{N}\sum_{l=2}^{N}\left[(N+1-k)(N+1-l)\cdot\left(\sqrt{(k-1)^2+(l-1)^2}\right)^{-\alpha}\right] \quad \text{(S6)}$$

From the above equation, we have:

$$\bar{\rho}_A < \frac{4\rho_1}{N^2(N^2-1)}\sum_{k=1}^{N}\sum_{l=2}^{N}\left[N^2\cdot\left(\sqrt{N^2+N^2}\right)^{-\alpha}\right] \cong 2^{2-\frac{\alpha}{2}}\rho_1 N^{-\alpha} \triangleq c_1N^{-\alpha}$$

and

$$\bar{\rho}_A > \frac{4\rho_1}{N^2(N^2-1)}\sum_{k=1}^{N/2}\sum_{l=2}^{N/2}\left[(N+1-k)(N+1-l)\cdot\left(\sqrt{(k-1)^2+(l-1)^2}\right)^{-\alpha}\right]$$

$$> \frac{4\rho_1}{N^2(N^2-1)}\sum_{k=1}^{N/2}\sum_{l=2}^{N/2}\left[\frac{N}{2}\cdot\frac{N}{2}\cdot\left(\sqrt{\left(\frac{N}{2}\right)^2+\left(\frac{N}{2}\right)^2}\right)^{-\alpha}\right] \cong 2^{-2-\frac{\alpha}{2}}\rho_1 N^{-\alpha} \triangleq c_2N^{-\alpha}$$

Recall $A = N^2$, we have: $c_2A^{-\alpha/2} < \bar{\rho}_A < c_1A^{-\alpha/2}$. Substitute into Eq. S1, we have:

$$-\frac{\ln\left(c_2A^{-\frac{\alpha}{2}}+A^{-1}\right)}{\ln A} < z_{\text{asym}} < -\frac{\ln\left(c_1A^{-\frac{\alpha}{2}}+A^{-1}\right)}{\ln A} \quad \text{(S7)}$$

As A goes to infinity, both sides of Eq. S7 converge to the below limit:

$$\lim_{A\to\infty}\frac{-\ln\left(cA^{-\frac{\alpha}{2}}+A^{-1}\right)}{\ln A} = \lim_{A\to\infty}\frac{\frac{\alpha c}{2}A^{-\frac{\alpha}{2}}+A^{-1}}{cA^{-\frac{\alpha}{2}}+A^{-1}} = \begin{cases}\frac{\alpha}{2} & \alpha < 2\\ 1 & \alpha \ge 2\end{cases} \quad \text{(S8)}$$

which is independent of c. Therefore, we have:

$$z_{\text{asym}} = \begin{cases} \frac{\alpha}{2} & \alpha < 2 \\ 1 & \alpha \geq 2 \end{cases} \quad \text{(S9)}$$

The triphasic curve of IAR

In the main text, we show that under the exponential correlation-distance function, IARs exhibit triphasic curves. As we have demonstrated above, IARs have asymptotic slopes of 1 under this scenario. This explains the third-stage steep increase of stability with area. Below we make some explanations on the first two stages of such triphasic curves.

We first consider a special scenario, in which between-patch correlation always equal ρ_1 regardless of distance (where $0 < \rho_1 < 1$). Following Eq. 1 in the main text, IAR can be expressed as:

$$I(A) = I_1 \cdot \frac{A}{(A-1)\rho_1+1} \quad \text{(S10)}$$

As shown in the Methods, the initial slope is given by: $z_{\text{ini}} = \log_2(2/(1+\rho_1))$, which lies between 0 and 1. As the area A goes to infinity, we have: $I(\infty) = I_1/\rho_1$. In other words, invariability increases with area at the beginning and converges to a constant when A is very large, i.e. the asymptotic slope of IAR is 0 (Supplementary Note Fig. 1a). This result can be understood more intuitively in an ecological context. Consider a landscape with regularly distributed square patches. The biomass dynamics of each local patch i (X_i) is governed by both environmental and demographic stochasticity: $X_i = n_0 + E + D_i$. Here, n_0 is the temporal mean biomass; E and D_i are random variables (mean: 0; variance: v_e and v_d), which represent, respectively, landscape-level environmental stochasticity and patch-level demographic stochasticity. We assume E and D_i are independent of each other, and D_i and D_j are also independent. Intuitively, we can expect that the effect of demographic stochasticity decreases with the area considered. So, as the area becomes large, the invariability of biomass should converge at some point that simply reflects the effect of

environmental stochasticity. In mathematical language, this writes: the invariability of one patch is: $S_1 = n_0^2/(v_e + v_d)$; invariability of two patches is: $S_1 = n_0^2/(v_e + v_d/2)$; … invariability of A patches is: $n_0^2/(v_e + v_d/A)$. Thus, IARs exhibit a faster increase with area at the beginning; the increase slows down, and the invariability converges to n_0^2/v_e when A is large.

Now we come back to the scenario of exponential correlation-distance functions. Within the area L^2, the between-patch correlation declines with distance very slowly (e.g. at a distance L, the between-patch correlation is $0.37\times\rho_1$, still in the same magnitude of ρ_1). Thus, if L is large, we can expect that IARs exhibit a fast increase at the beginning and followed by a "flat phase" near L^2. Note that because the between-patch correlation is often lower than ρ_1 (though always at the same magnitude with ρ_1), invariability at this "flat phase" is larger than $1/\rho_1$ (Supplementary Note Fig. 1b).

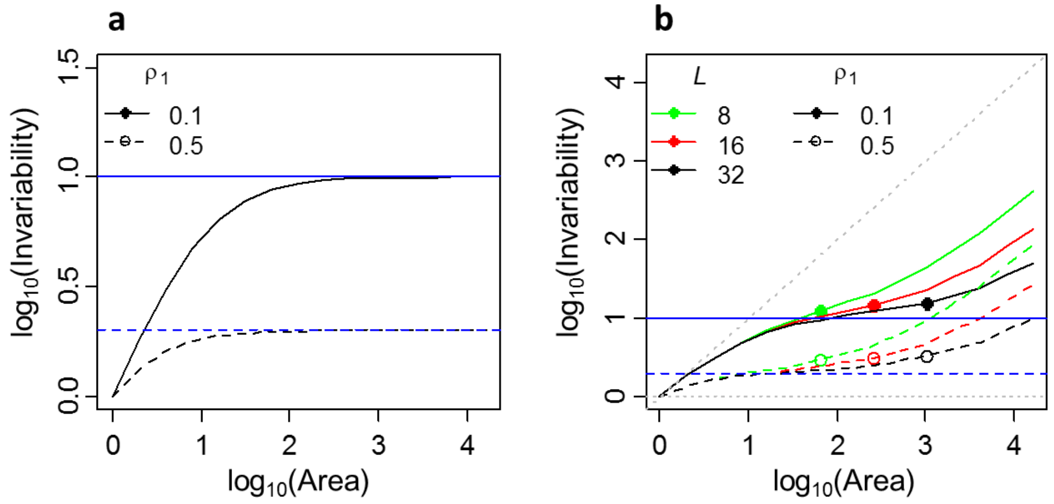

Supplementary Note Figure 1: IARs in two-dimensional landscapes. (**a**) IARs under a constant between-patch correlation (see Eq. S10 in Supplementary Note 2). Blue lines show horizontal lines corresponding to $1/\rho_1$. (**b**) IARs under exponential correlation-distance functions, same as in Fig. 1A. Blue lines show horizontal lines corresponding to $1/\rho_1$.

Supplementary Note 2: Sampling issues in IAR

In empirical analyses, the sampling scheme related to both spatial and temporal scales may affect the calculation of invariability and hence IAR. The main focus of this study is on spatial scale. Spatial scale can be represented by three aspects: extent, resolution, and sampling intensity. Since our study explores space continuously, the extent and resolution are represented by the largest and smallest area, respectively, on the x-axis of IAR. For the third aspect, i.e. sampling intensity, our paper has examined IARs on both continuous landscapes (i.e. flush grids), in our model and in the primary productivity data, and non-continuous ones (i.e. spatially separated grids due to incomplete sampling), in the bird data. In this Supplementary Note, we will explore the influence of sampling intensity on IAR with our theoretical model. As we will show, incomplete spatial sampling could potentially increase the slope of IAR.

Temporal sampling scheme might also influence the calculation of invariability and its scaling patterns, as previous studies suggested[1-3]. Temporal scale can similarly be represented by three aspects: observation length, sampling resolution, and sampling intensity. In this Supplementary Note, we will investigate the effect of observation length on IARs of bird biomass. As we will show, observation length can slightly alter the intercept and slope of IAR. However, we have not investigated the influences of sampling resolution and intensity in our study. As for the sampling resolution, our study has fixed it to one year due to both data limitation (e.g. bird biomass data is collected once per year) and research interest (e.g. we are interested in the interannual dynamics of NPP, not seasonal oscillations). As for the sampling intensity, we have fixed it to be annually. This is simply because we want to use most of available information in the data, given the relatively short time series (i.e. NPP data: 15 year; bird data: 21 years). This said, investigations into these aspects of temporal scales may be useful for future studies on IAR.

The effect of incomplete spatial sampling on IAR

In our models, we construct IAR based on a "full observation" of the landscape. However, in reality, field investigations may cover only a small proportion of the full landscape, for instance the North American Breeding Bird Survey in our empirical analyses. Here we explore the impact of incomplete sampling on IAR by constructing IAR based on a randomly sampled proportion of the landscape.

As in the main text, we consider a two-dimensional landscape (e.g. 128×128 grids), in which local ecosystem dynamics have identical temporal mean and variability and the between-patch correlation decays with distance following a power function: $\rho(d) = \rho_1 \times d^{-\alpha}$. We randomly sample a proportion of grids (e.g. 1/4, 1/16, 1/64, etc.) from the whole landscape, which can be regarded as the sampled area in empirical studies. We construct IARs based on these sampled grids, following similar procedures as in our empirical analyses of the bird data. Specifically, starting from one (sampled) grid, we increase the number of grids by including the closest neighbor (sampled) grids. We calculated the temporal invariability for each respective "area", thus generating an Invariability-Area Relationship. Note that just as for our analysis of BBS data, the "area" here is the sampled area, or the number of grids.

We found that incomplete sampling tended to increase the slopes of IARs (Supplementary Fig. 2). We rescale the sampled area (or number of grids) by multiplying it with the average "represented area" of each sample (i.e. 4, 16, 64, respectively), which represents the spatial extent of the sampling efforts (Supplementary Fig. 2). Note, however, this rescaling does not alter the slope between invariability and area on a log-log scale (but it does alter the intercept).

The effect of observation length on IAR

The observation length (i.e. the length of the census) may also affect temporal invariability and its spatial scaling. Because of the autocorrelation of population and ecosystem dynamics, temporal variability can increase with the observation length[1,2]. This may potentially affect the slope of IAR, especially if the strength of this effect varies across spatial scales.

With the bird data, we examined how observation length might affect IARs. We compared the intercept and slope of IARs during the period 1990-2010 (i.e. results in the main text) to that during 1990-2000. Results showed that under the shorter observation period (i.e. 1990-2000), the intercept of IAR is higher (shorter vs. longer period: 1.15 vs. 1.02), but the slope is lower (0.4 vs. 0.45) (Supplementary Fig. 3a). Also, under the shorter observation period, correlation decays with distance more slowly (-0.40 vs. -0.55) (Supplementary Fig. 3b). Species-level IARs generally exhibit higher intercepts under the shorter observation period, but no systematic increase or decrease for the slope (Supplementary Fig. 3c,d).

Therefore, a longer observation period can decrease the intercept of IAR, but its effect on the slope of IAR is context dependent. To better understand these patterns, future models should incorporate autocorrelation in environmental fluctuations (e.g. red noise) and investigate how it interacts with local population dynamics and dispersal to regulate IARs.

The effect of grain size on correlation-distance relationship

In our model, we have assumed two correlation-distance functions to calculate IARs on a two-dimensional landscape. These correlation-distance functions describe how the correlation between two unit-size patches changes with distance. Here we show that the between-patch correlation also depends on the grain size.

To illustrate this, we consider four patches. Two of them (X_1 and X_2) are neighbors and located around point X (biomass are denoted as N_{x1} and N_{x2}, respectively), and the other two (Y_1 and Y_2) are neighbors and located around point Y (biomass: N_{y1}, N_{y2}) (see Supplementary Note Figure 2). The distance between X and Y (d_{xy}) are much larger than unit length (which is the distance between X_1 and X_2 or Y_1 and Y_2). The correlation between patches X_1 and X_2 or between patches Y_1 and Y_2 is ρ_1, and the correlation between patches at location X and Y (e.g. X_1 and Y_1) is ρ_{dxy}. In other words, at a grain size of 1, the between-patch correlation at a distance d is ρ_{dxy}. Now we calculate the between-patch correlation at a distance d when the grain size is 2.

In doing so, we regard the two patches X_1 and X_2 as one large patch X_{12} (biomass: $N_{x12} = N_{x1} + N_{x2}$) and Y_1 and Y_2 as one large patch Y_{12} ($N_{y12} = N_{y1} + N_{y2}$). We first calculate the variance of the patch X_{12} (or equivalently, Y_{12}): $\mathrm{Var}(N_{x12}) = \mathrm{Cov}(N_{x1} + N_{x2}, N_{x1} + N_{x2}) = 2\sigma^2(1 + \rho_1)$. We then calculate the covariance of biomass dynamics between X_{12} and Y_{12}:

$$\mathrm{Cov}(N_{x12}, N_{y12}) = \mathrm{Cov}(N_{x1} + N_{x2}, N_{y1} + N_{y2})$$
$$= \mathrm{Cov}(N_{x1}, N_{y1}) + \mathrm{Cov}(N_{x1}, N_{y2}) + \mathrm{Cov}(N_{x2}, N_{y1}) + \mathrm{Cov}(N_{x2}, N_{y2})$$
$$= 4\sigma^2\rho_{dxy}$$

So, the correlation coefficient between patches X_{12} and Y_{12} is: $\mathrm{Corr}(N_{x12}, N_{y12}) = \mathrm{Cov}(N_{x1} + N_{x2}, N_{y1} + N_{y2}) / [\mathrm{Var}(N_{x12}) \times \mathrm{Var}(N_{y12})]^{0.5} = 2\rho_{dxy} / (1 + \rho_1)$. Thus, the between-patch correlation at a distance d for a grain size of 2 is: $2\rho_{dxy} / (1 + \rho_1)$. This is always larger than that for a grain size of 1 (i.e. ρ_{dxy}), unless ρ_1 equals 1.

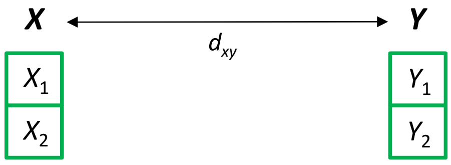

Supplementary Note Figure 2: Four patches on a two-dimensional landscape. The patches X_1 and X_2 are neighbors and located around point X, and the patches Y_1 and Y_2 are neighbors and located around point Y.

Supplementary Note 3: Ecological determinants of IAR

Our theoretical and empirical analyses show that the shape and slope of IAR are mainly determined by patterns of spatial synchrony of ecosystem dynamics. As spatial synchrony increases (e.g. a higher local correlation or a slower correlation decay with distance), invariability increases with area more slowly (Supplementary Fig. 1). In our model, two correlation-distance functions ("short-tail" and "long-tail") are investigated. Although very simple, they seem to capture the spatial synchrony patterns of plant and bird communities at continental scales, which in turn explain the patterns of IARs in these two systems (Figs. 2 and 3).

Previous studies have highlighted several factors that regulated spatial synchrony. In general, spatial synchrony in populations or ecosystem dynamics increases with environment correlation, dispersal, and community similarity[4-7]. Trophic interactions may also increase spatial synchrony through phase locking[8]. In our model, these factors are incorporated implicitly into scenarios of spatial synchrony (e.g. correlation-distance functions). However, a further understanding of IAR will require mechanistic models that incorporate lower-level abiotic and biotic processes (e.g. environment, dispersal, etc.) in a dynamical landscape, and clarify how they interact and regulate spatial synchrony and consequently IAR. In particular, the relative importance of these factors is likely scale dependent, and one important but challenging task is to tease apart their effects across scales. Such theoretical insights will advance our understanding on empirical patterns of IAR, e.g. the triphasic curve in Fig. 2.

Finally, IAR can be affected by spatial heterogeneity. This was demonstrated by our empirical results. For both primary productivity and bird biomass, we had calculated StARs iteratively using different starting points. Results showed that different starting points generated considerable variations among the resulting IARs, although they all reached the same end (i.e.

the area and invariability of the whole region) (Figs. 2&3). In our model, we have assumed a homogeneous landscape, i.e. the mean and variance of local biomass are identical across patches; see main text. However, despite ignoring spatial heterogeneity, our model predictions were well supported by the data (e.g. exponential correlation-distance relationships generate triphasic IAR (comparing Fig. 1 vs. Fig. 2), and IAR slope decreases with local correlation but increase with the exponent of power-law decay (comparing Supplementary Fig. 1 vs. Fig. 3)). This said, it would be useful for future theoretical work to clarify how spatial heterogeneity may affect the scaling of invariability.

Supplementary Note 4: IAR as an indicator for regime shifts

In spatially structured systems, spatial correlation has been used as an early warning signal for regime shifts[9]. Prior to a shift, spatial correlations between neighboring and distant patches are bound to increase[9]. Because IARs are shaped by patterns of spatial synchrony, regime shifts might also be detected by monitoring IARs. In this note, we develop a metapopulation model to illustrate the potential of IAR as an indicator for regime shifts.

Our model consists of N patches arranged in a ring and connected by dispersal (N = 512 in our simulations). The model setting follows Dakos et al.[9]. Within each patch, the dynamics of population biomass are described by an overharvesting model[10]. As the harvesting rate increases, the population shifts from an underexploited state with high biomass to an overexploited state with low biomass. Each patch is connected to its closest m neighbors via dispersal. Random environmental perturbations were added in each patch, enabling us to simulate a monitoring of IARs. Thus, our model reads[9]:

$$\frac{dx_j}{dt} = r_i x_j \left(1 - \frac{x_j}{k}\right) - c\frac{{x_j}^2}{{x_j}^2+1} + d\left(\sum_{0<|i-j|\leq\frac{m}{2}} x_i - m x_j\right) + \sigma_j B dW_j \qquad \text{(S11)}$$

Here x_j is the population biomass in patch j. r_j is the maximum growth rate for patch j, which is considered to have spatial heterogeneity (in our simulation, $r_j \sim U[0.6, 1]$). k is the carrying capacity (k = 10). c is the harvesting rate, which acts as a bifurcation parameter between underexploited and overexploited states. d is the dispersal rate (d = 0.5). m is the number of connected patches (m = 16). σ is the standard derivation of random noise (σ = 0.02). B is a symmetric matrix specifying the spatial correlation of random perturbations. Specifically, B^2 is the correlation matrix of noises that satisfies: $B^2(i,i) = 1$ and $B^2(i,j) = \rho_1 e^{-\frac{|i-j|}{L}}$ ($\rho_1 = 0.05$ and $L = 8$).

The overharvesting model is a minimal model that can have alternative stable states[10], and its spatial extension (i.e. equation S11) also exhibits alternative stable states[9]. In the absence of dispersal and environmental perturbations, a local population shifts from an underexploited state to an overexploited one as the harvesting rate (c) increases (Supplementary Figure 4a). Due to dispersal between patches and despite the fact that not all patches reach their own tipping point at the same time, a global tipping point exists at which all populations collapse onto the overexploited state. Prior to the shift, a slower speed of recovery following perturbations together with a spatial coupling allows perturbation to transmit to distant patches, thus increasing the system's spatial synchrony and altering the shape of IAR.

With this model, we explore how IAR changes as the harvesting rate (c) increases and approaches its critical value at the global tipping point (around $c^* = 2.0615$ for the set of parameters used in our simulations). Away from the regime shift, IAR exhibits a triphasic curve, with steeper increases in invariability at both small and large scales (Supplementary Fig. 4b). Approaching the global tipping point, local population dynamics exhibit larger variability, as previous studies showed[11]. This causes the intercept of IAR (i.e. local invariability) to decrease prior to the regime shift (Supplementary Fig. 4b, d). Moreover, a slowing down of the dynamics in each patch allows perturbations to propagate across space, increasing the correlation between neighboring and distant patches[12]. As a consequence, the initial and final slopes of IAR both decrease (Supplementary Fig. 4c,e,f) so that the triphasic shape gradually diminishes (Supplementary Fig. 4c). Remarkably, because spatial correlations do not increase in the same way at all scales but gradually propagate through space, the decrease in the initial slope of IAR occurs first, followed by, close to the shift, a decrease in the final slope (Supplementary Fig. 4e,f). Thus, monitoring IAR could add to the arsenal of early warning indicators of regime shifts.

Supplementary References

1. Pimm, S. L. & Redfearn, A. The variability of population densities. *Nature* **334**, 613-614 (1988).
2. Inchausti, P. & Halley, J. Investigating long-term ecological variability using the global population dynamics database. *Science* **293**, 655-657 (2001).
3. Halley, J. M. How do scale and sampling resolution affect perceived ecological variability and redness? In *The Impact of Environmental Variability on Ecological Systems* (Springer Netherlands, 2007).
4. Liebhold, A., Koenig W. D. & Bjørnstad, O. N. Spatial synchrony in population dynamics. *Annu. Rev. Ecol. Evol. Syst.* **35**, 467-490 (2004).
5. Ranta, E., Lundberg, P. & Kaitala, V. *Ecology of populations* (Cambridge Univ. Press, 2006).
6. Lande, R., Engen, S. & Sæther, B. E. Spatial scale of population synchrony: environmental correlation versus dispersal and density regulation. *Am. Nat.* **154**, 271-281 (1999).
7. Wang, S. & Loreau, M. Biodiversity and ecosystem stability across scales in metacommunities. *Ecol. Lett.* **19**, 510-518 (2016).
8. Vasseur, D. A. & Fox, J. W. Phase-locking and environmental fluctuations generate synchrony in a predator-prey community. *Nature* **460**, 1007-1010 (2009).
9. Dakos, V., van Nes, E. H., Donangelo, R., Fort, H. & Scheffer, M. Spatial correlation as leading indicator of catastrophic shifts. *Theor. Ecol.* **3**, 163-174 (2010).
10. May, R.M. Thresholds and breakpoints in ecosystems with a multiplicity of stable states. *Nature* **269**, 471-477 (1977).
11. Scheffer, M. *et al.* Early-warning signals for critical transitions. *Nature* **461**, 53-59 (2009).
12. Lande, R., Engen, S. & Sæther, B. E. Spatial scale of population synchrony: environmental correlation versus dispersal and density regulation. *Am. Nat.* **154**, 271-281 (1999).
